# Performance of a diagnostic algorithm for fibrotic hypersensitivity pneumonitis. A case–control study

**DOI:** 10.1186/s12931-021-01727-7

**Published:** 2021-04-23

**Authors:** Sabina A. Guler, Eva Wohlfarth, Sabina Berezowska, Thomas K. Geiser, Lukas Ebner, Manuela Funke-Chambour

**Affiliations:** 1 Department of Pulmonary Medicine, Inselspital, Bern University Hospital, University of Bern, 3010 Bern, Switzerland; 2 Schlosspraxis Schwarzenburg, Schwarzenburg, Switzerland; 3 Institute of Pathology, University of Bern, Bern, Switzerland; 4 Department of Laboratory Medicine and Pathology, Institute of Pathology, Lausanne University Hospital and Lausanne University, Lausanne, Switzerland; 5 Department of Diagnostic, Interventional and Pediatric Radiology, Inselspital, Bern University Hospital, University of Bern, Bern, Switzerland

**Keywords:** Pulmonary Fibrosis, Environmental Exposure, Algorithms, Diagnosis, Alveolitis, Extrinsic Allergic

## Abstract

**Background:**

The differential diagnosis fibrotic hypersensitivity pneumonitis (HP) versus idiopathic pulmonary fibrosis (IPF) is important but challenging. Recent diagnostic guidelines for HP emphasize including multidisciplinary discussion (MDD) in the diagnostic process, however MDD is not comprehensively available.

We aimed to establish the diagnostic accuracy and prognostic validity of a previously proposed HP diagnostic algorithm that foregoes MDD.

**Methods:**

We tested the algorithm in patients with an MDD diagnosis of fibrotic HP or IPF (case control study) and determined diagnostic test performances for diagnostic confidences of ≥ 90% and ≥ 70%. Prognostic validity was established using Cox proportional hazards models.

**Results:**

Thirty-one patients with fibrotic HP and 50 IPF patients were included. The algorithm-derived ≥ 90% confidence level for HP had high specificity (0.94, 95% confidence interval [CI] 0.83–0.99), but low sensitivity (0.35 [95%CI 0.19–0.55], J-index 0.29). Test performance was improved for the ≥ 70% confidence level (J-index 0.64) with a specificity of 0.90 (95%CI 0.78–0.97), and a sensitivity of 0.74 (95%CI 0.55–0.88). MDD fibrotic HP diagnosis was strongly associated with lower risk of death (adjusted hazard ratio [HR] 0.10 [0.01–0.92], *p* = 0.04), whereas the algorithm-derived ≥ 70% and ≥ 90% confidence diagnoses were not significantly associated with survival (adjusted HR 0.37 [0.07–1.80], *p* = 0.22, and adjusted HR 0.41 [0.05–3.25], *p* = 0.39, respectively).

**Conclusion:**

The algorithm-derived ≥ 70% diagnostic confidence had satisfactory test performance for MDD-HP diagnosis, with insufficient sensitivity for ≥ 90% confidence. The lowest risk of death in the MDD-derived HP diagnosis validates the reference standard and suggests that a diagnostic algorithm not including MDD, might not replace the latter.

## Background

Interstitial lung diseases (ILD) lead to significant symptoms, physical impairment, and early mortality [1, 2]. Fibrotic hypersensitivity pneumonitis (HP) is an ILD subtype caused by an ongoing or repetitive exposure to an inhaled antigen in sensitised and susceptible individuals [1]. The identification of patients with HP is crucial, particularly to prevent ongoing exposure to the causative antigen. However, in chronic cases where an inciting antigen cannot be identified [3], distinguishing fibrotic HP from idiopathic pulmonary fibrosis (IPF) is challenging with considerable diagnostic overlap between these fibrotic ILD subtypes [4, 5]. Patients with IPF have a worse prognosis than patients with fibrotic HP [6], and the immunosuppressive therapy that is frequently used in HP can be detrimental for IPF patients [7]. This emphasizes the importance of the differential diagnosis fibrotic HP versus IPF.

Due to the heterogeneity in clinical presentation among patients with fibrotic HP and IPF there is no single diagnostic test for accurate discrimination. The recent diagnostic guidelines for HP emphasize the importance of multidisciplinary discussion (MDD), and include MDD in the proposed diagnostic approach [5]. However MDDs are time and resource consuming and not widely available [8], simpler, accurate tools for HP diagnosis are urgently needed. A diagnostic HP algorithm that foregoes MDD has been developed by Morisset and group, with consensus criteria that were identified by a structured Delphi approach [9].

The main objective of this case control study was to validate the algorithm for the differential diagnosis fibrotic HP versus IPF. We aimed to establish the diagnostic accuracy of the algorithm, and to determine the prognostic validity of the algorithm and MDD derived HP diagnoses.

## Methods

### Study population, setting, and measurements

Patients with fibrotic ILD are prospectively recruited within our ongoing cohort study (Swiss Ethics Committee, Bern, KEK 246/15 PB_2016-01524). For this retrospective case control study, we included consecutive patients who were diagnosed with either fibrotic HP (cases) or IPF (controls) in our MDD between 03/2013 and 11/2018. Patients were included if the necessary data for a confident MDD diagnosis was available, including exposure history, high resolution computed tomography (HRCT) chest scans, and cytological or histological results if bronchoalveolar lavage (BAL) or lung biopsy were performed. All patients were questioned regarding exposure to a potentially inciting antigen using a questionnaire customized to our local practice. Antigen exposures that were considered relevant for HP included mould, hay, agricultural dusts, bird feathers and droppings, and the use of a hot tub, swimming pool, or air humidifier.

Aiming to evaluate the diagnostic accuracy and prognostic validity of a fibrotic HP diagnostic algorithm [9], we included patients with IPF as a control group, since IPF is the most challenging differential diagnosis. We defined MDD diagnosis as our reference standard [1, 2, 5, 8]. As previously described, our MDD meetings include experienced chest radiologists, pathologists, and specialized ILD physicians [8, 10]. For the purpose of this study, a chest radiologist who was blinded for the diagnosis (LE) reanalysed the chest CT scans of all IPF and fibrotic HP cases.

Patient demographics and baseline characteristics were collected from the registry. All measurements were accomplished within 3 months of MDD diagnosis. Pulmonary function tests were conducted according to established protocols [11, 12]. We calculated the Composite Physiologic Index (CPI), which was developed to predict radiological extent of fibrosis by aggregating forced vital capacity (FVC), forced expiratory volume in 1 s (FEV1), and diffusing capacity of the lung for carbon monoxide (DLCO).[13] Serum IgG against specific antigens were measured in some patients, however results were not included in the proposed algorihm [9], and not considered for this study.

### Algorithm for the diagnosis of fibrotic HP

**Fig. 1:**
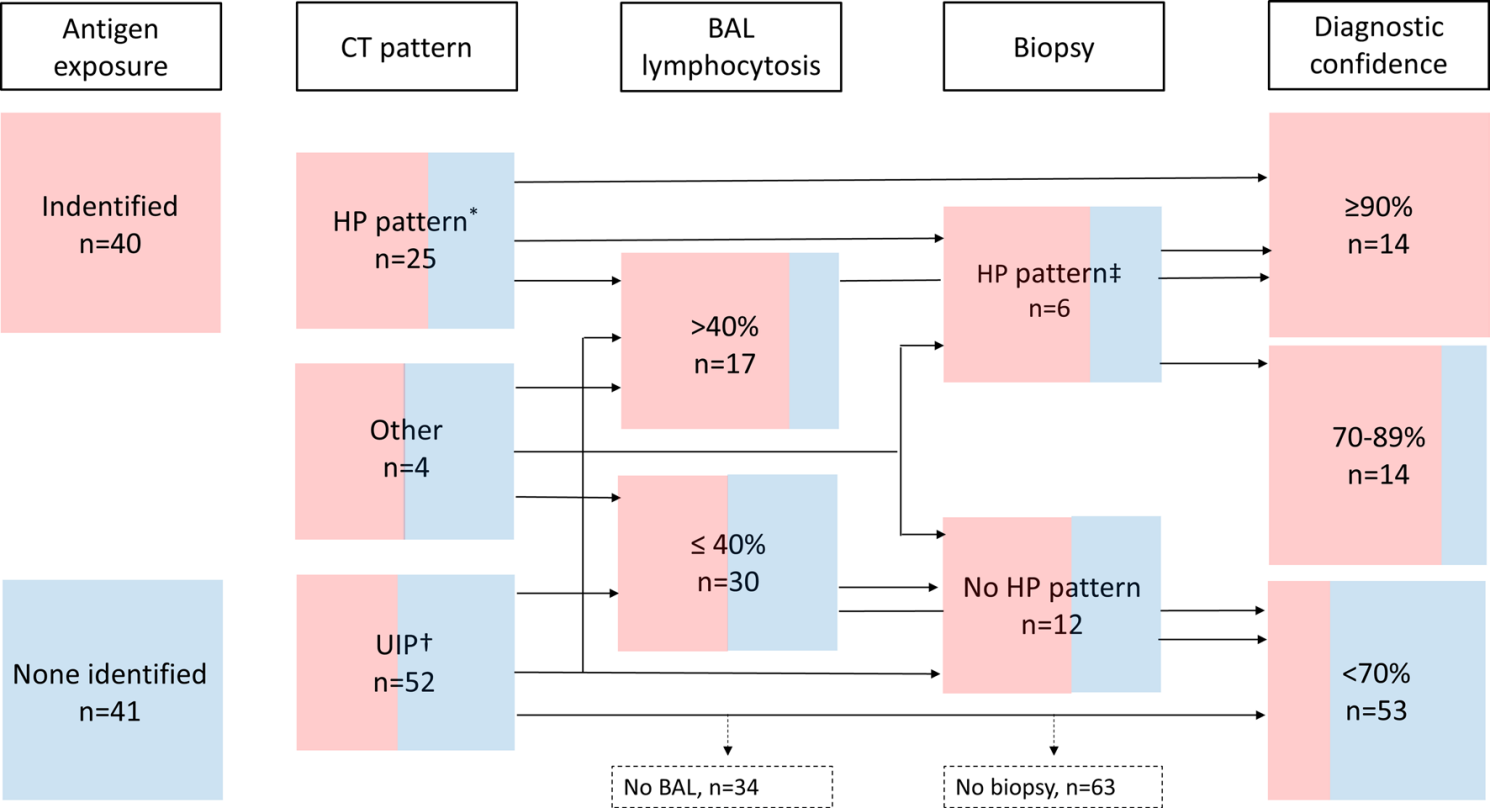
Diagnostic algorithm for fibrotic HP with patients stratified by presence (red) or absence (blue) of an identified antigen exposure. *Radiological HP pattern: Combination of mosaic attenuation, ground-glass and normal lung, or a combination of mosaic attenuation and signs of fibrosis. ^†^Radiological definite and probable usual interstitial pneumonia (UIP) based on the 2018 diagnostic criteria for IPF. ^‡^Pathological HP pattern: Chronic bronchiolocentric inflammation, poorly formed non-necrotizing granulomas, giant cells, airway-centred interstitial fibrosis, absence of an alternative diagnosis. *BAL* bronchoalveolar lavage, *HP* hypersensitivity pneumonitis, *IPF* idiopathic pulmonary fibrosis; *UIP* usual interstitial pneumonia

We applied a slightly adapted version of the HP diagnostic algorithm proposed by Morisset and group [9]. Every case was approached in a stepwise manner as outlined in the algorithm, starting with stratification by exposure to an inciting antigen (Fig. 1). As proposed, a HRCT pattern was considered typical for HP if either a combination of mosaic attenuation, ground-glass and normal lung, or a combination of mosaic attenuation and signs of fibrosis were present [9]. Usual interstitial pneumonia (UIP) and probable UIP were classified based on the most recent diagnostic criteria for IPF [2]. This in slight contrast to the Morisset algorithm, which included possible instead of probable UIP [14]. We also included *other* HRCT patterns as a third category, since in our cohort some HRCT pattern were neither classified as (probable) UIP nor as typical for HP. The threshold for lymphocytosis in BAL was set at 40% as suggested [9]. The histopathological features for HP included chronic bronchiolocentric inflammation, poorly formed non-necrotizing granulomas, giant cells, airway-centred interstitial fibrosis, and the absence of an alternative diagnosis [15]. We considered provisional diagnosis with high confidence (diagnostic confidence 70–89%) and a confident diagnosis (diagnostic confidence ≥ 90%) as diagnostically meaningful for the differential diagnosis fibrotic HP versus IPF. Low confidence diagnosis (diagnostic confidence 50–69%) was not evaluated for diagnostic test performance [9, 16].

### Statistical analysis

Baseline patient data are reported as mean (standard deviation) or median (interquartile range).

Diagnostic test characteristics with 95% confidence intervals (95% CI) were calculated for an algorithm derived diagnostic confidence ≥ 90% and ≥ 70% with an MDD diagnosis of fibrotic HP as reference standard. Sensitivity, specificity, positive and negative predictive values (PPV, NPV) were derived from two-by-two tables. Youden’s (J) index summarizes sensitivity and specificity, and is thus affected by disease prevalence, whereas diagnostic accuracy is the proportion of correctly classified cases among all cases, and not affected by disease prevalence. Based on the assumption that patients with a “true” diagnosis of fibrotic HP have a more favorable prognosis than patients with IPF [6, 17], we determined the prognostic validity of the MDD diagnosis and the algorithm derived diagnosis. Cox proportional hazards models were used to determine risk of mortality associated with baseline variables and fibrotic HP diagnoses (MDD versus algorithm derived). Adjusted models included radiological definite or probable UIP pattern, and CPI to account for potential confounding by ILD severity. Harrell’s C-statistic was used to indicate discrimination of the models. The proportional hazards assumption for all Cox regression models was tested based on weighted residuals. A two-sided p < 0.05 was used to indicate statistical significance. Data were analysed using R version 3.6.0 (R Foundation for Statistical Computing, Vienna, Austria) [18].

## Results

Thirty-one and 50 patients with an MDD diagnosis of fibrotic HP or IPF respectively were included in this study (Table 1). 78% of the cohort were men with a mean (standard deviation) age of 67 (10) years, and a moderately reduced FVC of 68 (19) %-predicted, and a DLCO of 51 (20) %-predicted. 40 patients (49%) reported exposures to potentially causative antigens (74% and 34% of MDD HP and IPF patients, respectively). A radiological probable or definite UIP pattern was present in 29% and 86% of MDD HP and IPF patients, respectively, with 68% of the HP and 8% of the IPF patients showing a radiological pattern compatible with HP. Of the patients with MDD diagnosed HP 26 (84%) had BAL available with BAL lymphocytosis present in 14 of these cases. BAL was available in 21 (42%) of MDD diagnosed IPF cases, with lymphocytosis in 3 patients. Surgical lung biopsy was available in 22% of IPF and 23% of HP patients.

**Table 1:** Baseline characteristics by MDD diagnoses

	Hypersensitivity pneumonitis n = 31	Idiopathic pulmonary fibrosis n = 50
Age, years	64.0 (10.7)	68.4 (9.5)
Sex, men	15 (48%)	48 (96%)
Ever smoker	12 (39%)	38 (76%)
Smoked pack years	20 (12.5–50)	30 (20–40)
Body mass index, kg/m^2^	27.9 (5.4)	27.2 (4.5)
FVC, %-predicted	73 (23)	65 (17)
FEV1, %-predicted	74 (18)	70 (17)
FEV1/FVC, %	103 (9)	108 (7)
DLCO, %-predicted	55 (20)	48 (19)
Composite Physiologic Index	42.0 (15.5)	48.5 (13.8)

*DLCO* diffusing capacity of the lung for carbon monoxide, *FEV1* forced vital capacity in 1 s, *FVC* forced vital capacity, *MDD* multidisciplinary discussion

### Diagnostic accuracy

A diagnostic confidence for fibrotic HP of ≥ 90% and a diagnostic confidence 70–89% were reached in 14 cases each (Fig. 1). The algorithm-derived ≥ 90% confidence level had a high specificity (0.94 [95% CI 0.83–0.99]) but low sensitivity (0.35 [95% CI 0.19–0.55]) for MDD diagnosis of fibrotic HP, with an overall insufficient test performance (J-index 0.29 [95% CI 0.03–0.53]). The algorithm-derived ≥ 70% confidence level showed higher overall test performance (J-index 0.64 [95% CI 0.34–0.85]) with improved sensitivity (0.74 [95% CI 0.55–0.88]), positive (0.82 [95% CI 0.63–0.94]), and negative predictive values (0.85 [95% CI 0.72–0.93]), with marginally lower specificity (0.90 [95% CI 0.78–0.97]), (Table 2).

**Table 2:** Diagnostic test characteristics for algorithm derived HP diagnoses

	Algorithm derived diagnostic confidence	
	≥ 90%	≥ 70%
Sensitivity (95% CI)	0.35 (0.19–0.55)	0.74 (0.55–0.88)
Specificity (95% CI)	0.94 (0.83–0.99)	0.90 (0.78–0.97)
J-index (95% CI)	0.29 (0.03–0.53)	0.64 (0.34–0.85)
PPV (95% CI)	0.79 (0.49–0.95)	0.82 (0.63–0.94)
NPV (95% CI)	0.70 (0.58–0.81)	0.85 (0.72–0.93)
Diagnostic accuracy	0.71 (0.60–0.81)	0.84 (0.74–0.91)

*CI* confidence interval, *HP* hypersensitivity diagnosis, *J-index* Youden’s index, *NPV* negative predictive value, *PPV* positive predictive value

### Prognostic validity

Over a median time of 16 months (interquartile range 6–30), 28 patients deceased.

Per one % increase in DLCO patients had a 5% decrease in risk of death (hazard ratio [HR] 0.95, 95%CI 0.92–0.98, *p* = 0.007). FVC %-predicted was borderline associated with mortality (HR 0.98, 95%CI 0.96–1.00, *p* = 0.07), and CPI was significantly associated with mortality (HR 1.06, 95%CI 1.01–1.10, *p* = 0.01). Patients with a definite/probable radiological UIP pattern had a 5.4 higher risk of death (95% CI 1.62–17.8, *p* = 0.006). Age, sex, smoking status, body mass index, identification of the inciting antigen, and availability of surgical lung biopsy were not associated with survival.

**Fig. 2:**
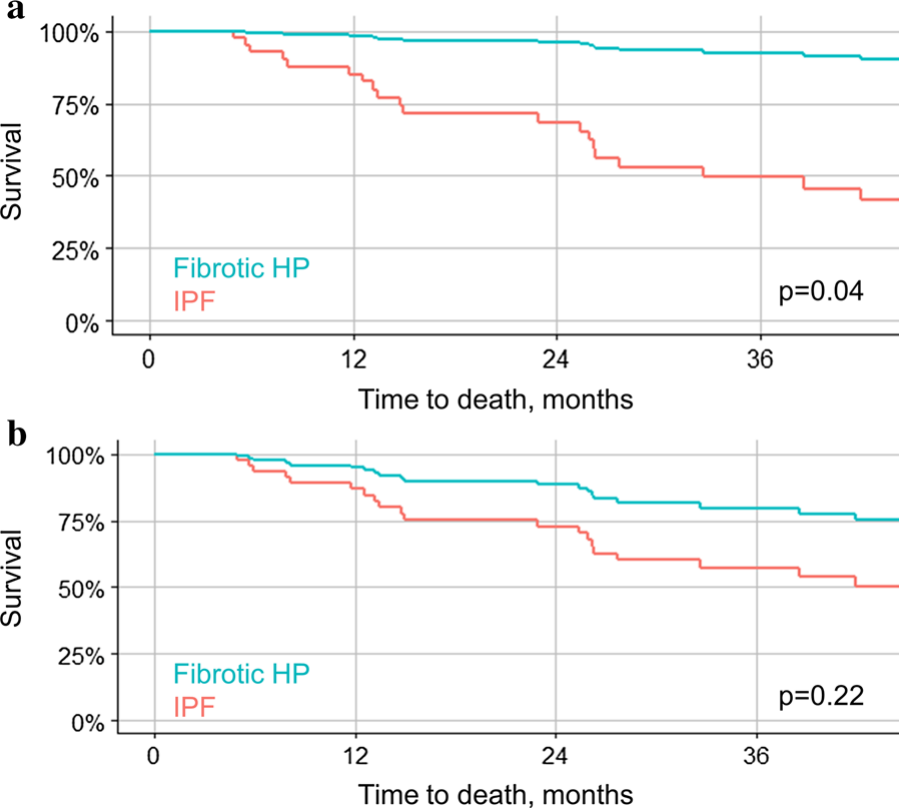
Survival by multidisciplinary discussion **a** and algorithm derived ≥ 70% diagnostic confidence **b** diagnoses for fibrotic HP versus IPF. Survival curves from Cox Proportional Hazard models adjusted for Composite Physiological Index and radiological pattern of definite or probable UIP pattern. *HP* hypersensitivity pneumonitis, *IPF* idiopathic pulmonary fibrosis, *UIP* usual interstitial pneumonia

MDD diagnosis of fibrotic HP was strongly associated with a lower risk of death on unadjusted analysis and with adjustment for potential confounding by disease severity (CPI) and a radiological UIP pattern (HR 0.10 [0.01–0.92], *p* = 0.04). The algorithm derived ≥ 70% confidence diagnosis showed a significant association with lower risk of death on unadjusted analysis, which lost statistical significance on adjusted analysis (HR 0.37 [0.07–1.80], *p* = 0.22). The algorithm derived ≥ 90% confidence diagnosis was not significantly associated with survival on unadjusted and adjusted analysis (HR 0.41 [0.05–3.25], *p* = 0.39), (Table 3, Fig. 2).

**Table 3:** Prognostic validity of MDD and algorithm derived HP diagnoses

	Unadjusted analyses			Adjusted for CPI and UIP		
	HR (95% CI)	*p*-value	C-index	HR (95% CI)	*p*-value	C-index
Multidisciplinary discussion diagnosis for fibrotic HP						
HP	0.06 (0.01–0.45)	0.006	0.68	0.10 (0.01–0.92)	0.04	0.76
Algorithm derived diagnosis for fibrotic HP						
≥ 90% confidence	0.16 (0.02–1.20)	0.07	0.58	0.41 (0.05–3.25)	0.39	0.72
≥ 70% confidence	0.14 (0.03–0.59)	0.007	0.65	0.37 (0.07–1.80)	0.22	0.72

*C-index* Harrell’s C-statistic, *HP* hypersensitivity pneumonitis, *HR* hazard ratio, *UIP* radiological usual interstitial pneumonia pattern

## Discussion

With this case control study, we validate a previously developed diagnostic algorithm for fibrotic HP [9], by assessing its diagnostic test performance in a cohort of patients with fibrotic HP and a control cohort of patients with IPF, using MDD as the reference standard. The algorithm derived diagnostic confidence ≥ 70% demonstrated a high specificity, acceptable sensitivity, and a diagnostic accuracy of 84%. An algorithm derived diagnostic confidence level ≥ 90% showed poor sensitivity and lower diagnostic accuracy (71%) with an expectedly higher specificity than the ≥ 70% confidence level. Overall, if a confidence level ≥ 70% was considered diagnostic for fibrotic HP, the algorithm performed well in our cohort, whereas diagnostic performance for the ≥ 90% threshold was insufficient.

MDD diagnosis of fibrotic HP showed a stronger association with survival than the algorithm derived diagnoses on unadjusted analysis, and with adjustment for disease severity and UIP pattern, which have been shown to impact survival in IPF and fibrotic HP beyond diagnosis [6, 17]. This observation strengthens the validity of MDD diagnosis as the reference standard in our cohort.

Identification of the inciting antigen is crucial for HP diagnosis and management. In patients with an identified antigen diagnostic confidence for HP is higher [9], the danger of IPF misclassification is lower [4], and prognosis is more favorable [3]. However, in less than 50% of patients an antigen can be identified [3]. We confirm that a systematic exposure assessment is helpful for antigen identification. With a questionnaire tailored to local practice we identified an antigen exposure in 74% of HP cases, but also in 34% of IPF cases, which underlines the low specificity of patient-reported antigen exposure, and the need for estimation of potential causality by the clinician [19]. Testing of specific serum IgG levels can confirm exposure to the antigen at some point in the patient’s life, without prove of causality to the ILD [5].

BAL lymphocytosis is important for HP diagnosis, either as a piece of information to the MDD or as a formal diagnostic criterion or integrated in an algorithm. However, the optimal BAL lymphocytosis cut-off still needs to be determined: A recent meta-analysis demonstrated an optimized sensitivity (71%) and specificity (68%) for a 21% lymphocytosis threshold [20], and a threshold of 20% has been proposed to decide if a surgical lung biopsy for suspected HP is needed [21]. Conversely, most ILD experts indicated that they find a threshold of 40% to be useful for the diagnosis of chronic HP [9], and experts contributing to the new diagnostic guidelines indicated that they find a threshold of 30% reasonable [5]. Likely different thresholds are valid for different positions of BAL in a diagnostic algorithm.

Recent guidelines suggested BAL, transbronchial cryobiopsy, and surgical lung biopsy for the diagnosis of fibrotic HP with very low confidence [5]. In 2 (6%) of our patients with an MDD diagnosis of HP and in 19 (35%) of the MDD diagnosed IPF controls, no surgical lung biopsy or BAL was performed. In the individual patient, the risk–benefit ratio of these invasive procedures is challenging to estimate. Aside from the diagnostic confidence before biopsy (which can be estimated with a diagnostic algorithm), the individual procedural risk likely determines the role of biopsy in the diagnostic algorithm. Consequently, a diagnostic HP algorithm might need tailoring for patients with advanced disease, important comorbidities, and frailty.

The multi-component diagnostic process emphasizes the importance of MDDs for diagnostic decision making in complex cases. The overlap between fibrotic ILDs and the heterogeneity within fibrotic HP and IPF might not be sufficiently accounted for by an algorithm in every case, and MDD discussion likely provides additional granularity for the assessment of complex cases. The prognostic validity of chronic HP MDD diagnosis in this study further supports this approach. In line, the recent diagnostic guidelines for HP confirm the central role of MDD in the diagnostic evaluation of patients with possible HP [5].

Acknowledging the limited availability of resources for MDD, a diagnostic algorithm might be valuable to pre-select typical cases where MDD discussion is less important. Future studies might investigate the implementation of diagnostic decision trees including the option of MDD discussion. Our findings suggest that an algorithm might be a valid tool for fibrotic HP diagnosis. The applicability of the algorithm might further be improved with adaptations according to local practice. Furthermore, future integration new diagnostic tools, e.g. blood-based biomarkers and genomic classifiers might improve the discriminative ability of diagnostic algorithms [22, 23].

Our local single centre study limits generalizability of the findings to populations with similar demographics and antigen exposure patterns. In our experience IPF is the most challenging and impactful differential diagnosis of fibrotic HP. However, our results do not apply for other differential diagnoses. A recent Japanese study reported the chronic HP algorithm to be useful in a heterogeneous ILD cohort, with potential avoidance of surgical lung biopsy in some of their ILD patients. However, 59% of cases were excluded from the algorithm, mainly because they did not have radiological features consistent with UIP or with HP [24]. By adding the “other radiological pattern” category to the algorithm, we ensured that every case was captured by the algorithm. Furthermore, prevalence of fibrotic ILD subtypes varies between cohorts, which can impact on PPV, NPV, and diagnostic accuracy. Sensitivity, specificity, and Youden’s index are not influenced by disease prevalence. Our adaptations to the proposed algorithm were minor, but necessary to include all cases and controls in the algorithm. With the application of a customized antigen exposure questionnaire and detailed interviews, we tried to capture all relevant exposures, however missing exposures are still possible. Incorporation bias is an inherent problem to many diagnostic test studies in ILD. The diagnostic pieces of the algorithm are also included in MDD discussions, which likely inflates the agreement between the two assessments. Furthermore, there is no “gold standard” for fibrotic HP diagnosis. We tried to account for this by demonstrating the prognostic validity of our reference standard.

## Conclusion

Current diagnostic guidelines for HP recommend a diagnostic approach that includes MDD [5]. In this study, we validate a proposed diagnostic algorithm for fibrotic HP that foregoes MDD. We demonstrate acceptable test performance of the ≥ 70% diagnostic confidence level compared to the reference standard MDD, and consequently endorse the incorporation of algorithms to fibrotic HP diagnosis. Due to the low sensitivity and potential danger of missing HP cases, we suggest not relying on the current algorithm’s ≥ 90% diagnostic confidence level for the differential diagnosis HP versus IPF. Algorithms likely need to be adapted to regionally distinct exposures, local practice, and availability of diagnostic tests, with prospective validation in corresponding cohorts. Furthermore, algorithms might not be sufficiently accurate for complex cases and are unlikely to replace MDDs entirely.

## Data Availability

The datasets used and analysed during the current study are available from the corresponding author on reasonable request.

## Abbreviations

BAL: Bronchoalveolar lavage
CI: Confidence interval
C-index: Harrell’s C-statistic
CPI: Composite Physiologic Index
DLCO: Diffusing capacity of the lung for carbon monoxide
FEV1: Forced expiratory volume in 1 s
FVC: Forced vital capacity
HP: Hypersensitivity pneumonitis
HR: Hazard ratio
HRCT: High resolution computed tomography
ILD: Interstitial lung disease
IPF: Idiopathic pulmonary fibrosis
J-index: Youden’s index
MDD: Multidisciplinary discussion
NPV: Negative predictive value
NSIP: Nonspecific interstitial pneumonia
PPV: Positive predictive value
UIP: Usual interstitial pneumonia
